# Modifying the Bass diffusion model to study adoption of radical new foods–The case of edible insects in the Netherlands

**DOI:** 10.1371/journal.pone.0234538

**Published:** 2020-06-11

**Authors:** Andrijana Horvat, Vincenzo Fogliano, Pieternel A. Luning

**Affiliations:** Department of Agrotechnology and Food Sciences, Food Quality and Design Group, Wageningen University and Research, Wageningen, The Netherlands; West Pomeranian University of Technology, POLAND

## Abstract

Developing new food products is a complex process. Even if a company performs new product development activities successfully, it is still uncertain if consumers will adopt the product. The Bass diffusion model has often been used to study product adoption. However, existing modifications of the Bass diffusion model do not capture the complexity of consumer food choice and they have limitations in situations where there is no sales data. To avoid these challenges, the system dynamics approach can be employed. This paper aimed at extending the existing system dynamics Bass diffusion model to investigate the dynamic adoption process of insect-based food from a consumer research perspective. We performed a structured review of the literature on edible insects to build the model. The model was used to study adoption of the product amongst consumers in the Netherlands. Simulations revealed that diffusion of a radical innovation, such as an insect-based burger, can proceed for many years before there are observable adopters in the total population, under the currently reported practices in the Netherlands. Expanding awareness of this innovation requires many decades, which can be quickened by developing strategies aimed at increasing word-of-mouth. Nevertheless, the low likelihood to adopt such food remains a challenge towards full adoption, even when the sensory quality of products is improved. To fully explore how to improve the diffusion outcome of edible insects, more knowledge on mechanisms related to positive and negative word-of-mouth, and adoption of insect-based burgers by people who initially reject them, is needed. Our study demonstrated that system dynamics models could have potential in designing new food product strategies in companies, as they facilitate decision-making and uncover knowledge gaps.

## Introduction

Development of new food products is one of the most important activities in food companies, as successful new products contribute substantially to the growth of a company [1]. Even if a company performs new product development (NPD) activities successfully, it is still uncertain if a product will be adopted among consumers, which makes NPD a risky process [1]. NPD is a complex process, since various company functions (e.g., marketing, R&D, sales, production) need to work together to establish product attributes, produce the product, and launch it on the market [2]. Moreover, additional complexity arises due to changes in consumers’ needs and preferences, which are a result of a multitude of person-, product- and environment-related factors [3]. These factors enhance uncertainty whether consumers will adopt a product. Since consumers have the final verdict in new product adoption, understanding their needs and preferences has been a recommended approach to successful NPD [4].

Once case where uncertainty of product adoption is high is insect-based food, which has been promoted in the Western world as an alternative to meat protein sources. Although this type of food is common in some parts of the world, such as China and Africa, many consumers in the Western world are still reluctant to adopting it [5]. The Netherlands have been at the forefront of insect-based food business in the Western world [6, 7], where insect-based food has been promoted since the late 1990s [8]. For example, in Dutch supermarkets, burgers containing up to 15% of ground insects are sold regularly [9, 10]. However, the concern if Dutch people are ready to adopt such radical innovation remains.

To understand the potential of a new product being adopted, researchers have been using various tools and measures to study consumers’ preferences (see [11] for a review of tools and methods). For example, researchers have been studying, amongst others, consumers’ “purchase intent”, “willingness to try”, and “food neophobia” (e.g., review [12]). These tools aim at studying a multitude of person-related factors (psychological factors and socio-demographic characteristics) and food product attributes in relation to consumers’ food choice. Measures like “willingness to try” and “purchase intent” give invaluable guidelines to develop effective product design and product positioning strategies. These types of approaches increase the understanding of product adoption from a “detail complexity” perspective, which is a complexity where many variables and their interconnections are considered [13]. However, these approaches give limited insight in the evolvement of product success over a longer period [14], as they do not consider consumer product adoption from a dynamic perspective.

There has been a stream of research dedicated to studying product adoption from a dynamic perspective, i.e., if a product will be adopted over time, stemming from the diffusion of innovations paradigm. The diffusion of innovations (DoI) paradigm has been established as a common way of researching the spread of a new product in the marketplace [15]. In the 1960s, DoI was used to develop the mathematical Bass diffusion model, which was employed to analytically study new product adoption on the market [16, 17]. The original Bass diffusion model consisted of a limited number of variables, and the decades following its development saw a rise of literature dedicated to expanding it (e.g., [18]). However, the Bass diffusion model requires sufficient sales data (e.g., of the existing or analogous product) to estimate the model parameters [19]. Obtaining data of processed foods can be particularly challenging, especially in situations when a new to the world food product (e.g., radical food such as edible insects) is being developed and no similar products exist on the market that could be used to estimate the parameters of the analytical Bass diffusion model [20, 21]. In such situations, simulation modeling can be useful to study complex problems, such as product adoption over time [22].

The adoption process is a problem that has commonly been approached by a simulation modelling approach called system dynamics (SD). System dynamics simulation models focus on dynamic complexity, wherein one investigates causal relationships between variables, which lead to different types of behaviour over time. Instead of using only sales data to estimate model parameters, other appropriate data can be used to formalize the model, such as data on customer choices (e.g., [23]). SD modelling is a way to simplify reality, making it easier to comprehend, with the purpose of testing possible consequences of various strategies [16], such as promotion intensity or product quality level. SD models are not predictive, but descriptive models [16, 24, 25]. One chooses to build an SD model when the aim is to improve general understanding of a dynamic problem by studying the patterns of change (i.e. the shapes of the curves over time that result from many different model simulations), and to identify knowledge gaps and guide future research efforts [25]. System dynamics, coupled with the diffusion of innovations paradigm, has been used to study a wide range of adoption problems (e.g., improved maize seed [26], alternative fuel vehicles [27], cell-phones [28], renewable energy [29], golf clubs [30], application of a product adoption model for pricing strategy [31], medical technologies [32]). However, to the best of our knowledge, the SD approach has not been adopted to study the adoption of radical new foods by consumers.

With this paper, we aimed at extending the existing SD Bass diffusion model to develop and simulate adoption of a radical new food product. We focused on the topic of insect-based burgers in the Netherlands to investigate the possibility of an SD approach to capture and study the dynamic diffusion process of this radical food innovation among consumers, and to identify knowledge gaps that could guide future research.

### Theoretical background

Since the diffusion of innovations paradigm and the Bass diffusion analytical model are the foundation for complexity modelling of new product diffusion [15–17], we used them to explore the topic of insect-based food adoption in the Netherlands.

### The diffusion of innovations paradigm (DoI)

The diffusion of innovations (DoI) paradigm is the social process of communicating a subjective evaluation of an innovation from person to person [33]. The outcome of the diffusion of an innovation is plotted as a cumulative number of adopters over time, in which case the plotted curve reveals an S-shaped growth [16, 33–34].

DoI consists of four main elements: 1) an innovation, 2) communication of the innovation through a certain channel, 3) the time over which this happens, and 4) a social system in which this occurs. An innovation is characterized by its newness to an individual, and not by its newness to the world. Communication channels are means of exchanging information about an innovation between an individual who has knowledge of, or experience, with the innovation, and an individual who has no knowledge or experience with it [29]. Diffusion occurs over a certain time, which is needed for individuals to communicate through the channels [18, 35]. Lastly, these individuals are part of a social system with certain boundaries, within which innovation diffusion occurs [33]. The time an individual takes to go through the process of adopting an innovation is affected by perceived attributes of the innovation, communication channels (e.g., mass media, interpersonal), the nature of the social system (e.g., norms), the extent of promotion efforts, and the type of innovation-decision [33]. Innovation decisions are distinguished based on who makes the decision and if it is made freely. There are three types of innovation-decisions: optional (made by an individual), collective (made collectively), and authority (made by individuals in positions of power for the entire social system) [33].

### Theoretical background on the Bass diffusion model of innovation adoption

There is an extensive body of literature on DoI in the marketing field. The main catalyst of this development was a new product growth model developed by Bass [17, 18]. The Bass diffusion model can be represented with the following mathematical equation [17]:

$$\frac{dN(t)}{dt}=p[m-N(t)]+\frac{q}{m}N(t)[m-N(t)],$$ (1)

where N(t) is the cumulative number of adopters at time t, m is the total population or potential adopters, p is the coefficient of innovation, and q is the coefficient of imitation.

The original Bass diffusion model has a fixed population of potential adopters and it assumes that they are influenced by mass media and word-of-mouth communication, which are affected by the coefficient of innovation, and the coefficient of imitation, respectively. These coefficients are estimated from existing sales data of a product, or by using historical sales data of an analogous product. The original Bass diffusion model underwent multiple extensions in the last decades. For example, marketing mix variables (e.g., price, advertising, distribution), market potential changing over time due to the growth in households and population, in the number of retailers selling the product, or due to income distribution have been considered [18, 19]. Moreover, additions like supply restrictions, impact of market and product characteristics and adoption of successive generations of products have been noted [18].

The Bass diffusion model is also common in the SD field, since it has been translated from its algebraic form to the SD form. System dynamics is a methodology to study complex problems, which have two main characteristics. Firstly, the problems are dynamic; they involve quantities that change over time, which can be expressed as graphs of variables over time [36]. Secondly, the problems involve the notion of feedback [36]. The main sources of dynamics in a system are cause-effect relationships between variables in the system, in the form of positive and/or negative feedback loops [16, 36]. SD models are used to study patterns of behaviour or graphs of variables over time, and not for point prediction. They are descriptive models that allow comparison of the difference in patterns of behaviour among various scenarios [16, 24, 25].

The SD Bass diffusion model distinguishes two feedback mechanisms, namely market adoption or adoption through mass media (external influence), and word-of-mouth (internal influence) [16, 18, 37]. They act as two different communication channels. The external influence serves the purpose of “seeding” the market with early adopters, who then initiate word-of-mouth in the population among potential adopters [38]. The Bass diffusion model represents adoption of an innovation among members of a closed system [16, 17]. It belongs to the group of aggregate models where consumers are studied as a perfectly mixed collection of individuals with an aggregated behaviour, instead of representing individual behavioural characteristics of consumers [15, 39]. Therefore, numerical values of variables correspond to average values of the whole social system that is studied. The dynamic behaviour of the basic Bass diffusion model is represented by the S-shaped growth curve of a cumulative number of adopters over time, which is also characteristic for DoI.

Although both the marketing and SD field have a long tradition of research based on the Bass diffusion model, the existing model adaptions do not support studying adoption of a radical food product by consumers. In the marketing field, there has been a concern about the applicability of the hypotheses from the general diffusion research to consumer behaviour [40]. Food choice is affected by changes in consumers’ needs and preferences, resulting from a multitude of person-, product and environment-related factors [3]. Much of marketing diffusion of innovations research is on consumer durables and telecommunications (e.g., review by Meade and Islam [19]; the authors of this paper also performed a review of scientific papers on the Bass diffusion model on Scopus from 2005 until 2019). On the other hand, adoption of new foods by consumers has also not been studied with the SD approach. Therefore, there is a need to adapt the Bass diffusion model to the current literature on consumer adoption of insect-based food. To underline, there are two main reasons for making conceptual additions to an existing Bass diffusion model:

1. The majority of the diffusion of innovations research from 1970 until 2019 is on consumer durables, and not related to consumer non-durables such as food;
2. The existing non-SD Bass diffusion models usually contain variables that are related to marketing mix (e.g., price, distribution, promotion), which are not common in consumer research literature on edible insects.

Moreover, existing non-SD Bass diffusion models have limitations in situations where there is no existing sales data to estimate coefficients of imitation and innovation, such as in the case of insect-based food [19–21]. The SD modelling and simulation approach can overcome the lack of numerical data by focusing on underlying mechanisms leading to dynamic behaviour, such as food adoption, which can facilitate formalization and validation of such models [41]. Therefore, this paper will focus on studying insect-based food adoption by employing the SD approach.

### Conceptual framework for insect-based food adoption, resulting from theoretical background

**Fig 1:**
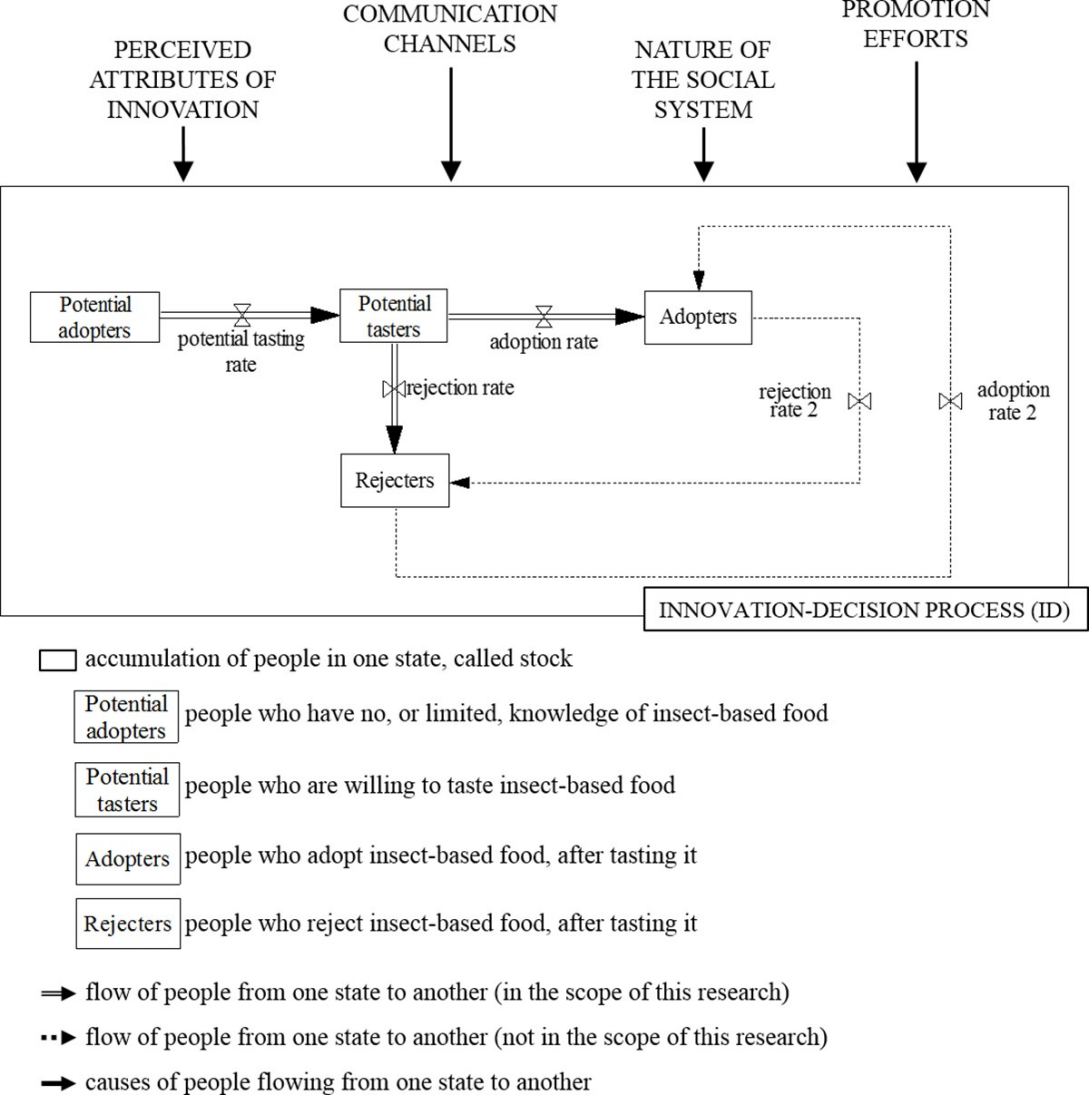
Framework to analyse insect-based food adoption literature and to build the model, based on the diffusion of innovations paradigm [33] and the Bass diffusion system dynamics model [16].

Based on the DoI paradigm and the Bass diffusion SD model, a conceptual framework (Fig 1) was developed to analyse current insect-based food adoption literature and to extend the existing SD model for adoption of insect-based food. The conceptual framework in Fig 1 has four stocks (“Potential adopters”, “Potential tasters”, “Adopters” and “Rejecters”) and five flows (“potential tasting rate”, “adoption rate”, “rejection rate”, “adoption rate 2” and “rejection rate 2”). Stocks represent accumulations of the Dutch population in different adoption phases. Flows represent the rates with which people move from one stage of the decision process to another. The flows are determined by any of the four main factors: perceived attributes of innovation, communication channels, nature of the social system, and promotion efforts. The framework in Fig 1 was developed from a consumer perspective, which implies an optional innovation-decision type, where decisions are not made collectively, but by individuals [33].

The basic Bass diffusion model has only two stocks and one flow (“Potential adopters”, “Adopters” and “adoption rate”). However, DoI suggests that people usually become adopters or active rejecters of an innovation only after they have had a chance to try it [33]. Therefore, two more stocks were added to make a distinction between people who merely decide to try insect-based burgers and the ones who decide to adopt it as part of their diet, or reject it, after tasting it. The stock of “Potential tasters” represents the population willing to try insect-based food. The stock of “Adopters” corresponds to the population that is likely to adopt a certain insect-based product after tasting it. In this respect, it is worth to notice that according to Tan et al. [5] more than 20% of Dutch consumers have tasted insects in past situations.

### Building the model

In this study, building the SD model is divided into two stages: 1) a qualitative, to perform literature review and develop an SD model structure, and 2) a quantitative, to formulate model equations and to perform model simulations. The qualitative stage resulted with a stock and flow diagram, which was the basis for the quantitative stage. The model was built using Vensim DSS software, Version 6.4b [42].

To establish model validity, multiple SD model verification and validation tests were performed. Theoretical structure confirmation test verified if the stock and flow structure is consistent with the knowledge about the system [43, 44]. For this purpose, a structured literature review of literature on edible insects obtained from Scopus database was performed. To assess the performance of the theoretical structure test, information obtained from the literature review and the structure of the stock and flow diagram was checked for consistency, i.e., if the causal relations and dynamic variables extracted from the literature are present in the stock and flow model. Only information related to the framework in Fig 1 was extracted from literature.

Once the model was formulated, dimensional consistency, and possible numerical errors because of inappropriate numeric integration and step size, were assessed [43]. To assess the performance of these tests, the software needed to show no errors in the model. Moreover, units of each parameter and variable needed to pass the conceptual parameter-confirmation test, i.e., the units needed to have a meaning in real life [43]. Moreover, sensitivity analysis, and extreme conditions tests were performed [16, 43] (see S1 File). Lastly, pattern behaviour test was performed [44]. To pass this test, the model needed to show S-shaped behaviour, while reaching the value of the variable “average familiarity of the population” of approximately 20% in the year 2015 [5, 16, 17].

### Developing the stock and flow diagram

The proposed framework (Fig 1) shows the boundary of the system, which was used for the literature review on insect-based food adoption to uncover relevant variables and their causal relations. More specifically, the boundary of the system is defined by the innovation-decision process (ID), as the backbone of the model, and the four factors—perceived attributes of innovation, communication channels, nature of the social system, and promotion efforts. As such, the model represents an overview of factors relevant under the proposed framework, with the focus on variables that affect the system dynamically.

#### From potential adopters to potential tasters

**Fig 2:**
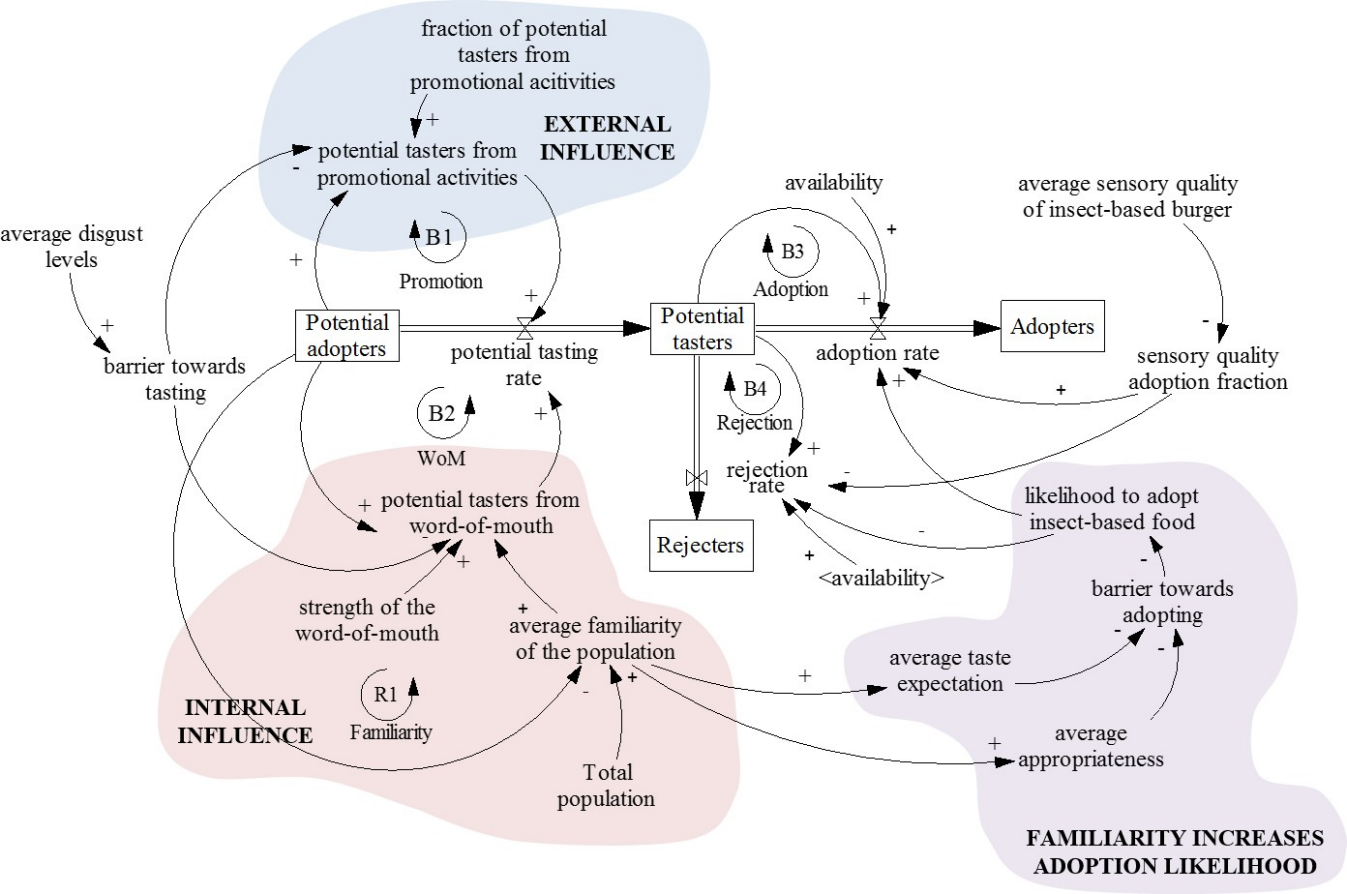
Stock and flow diagram of insect-based food adoption in the Netherlands. The 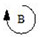 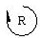 rate with which “Potential adopters” become “Potential tasters” of insect-based food depends on external influence and internal influence on “Potential adopters”. The rate with which “Potential tasters” become “Adopters” or “Rejecters” depends on their familiarity with insect-based food, which affects adoption likelihood, and average sensory quality of insect-based burger. The running model is in S1 Dataset. (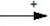 positive causal influence–other things being equal, an increase in variable A causes an increase in variable B, or a decrease in variable A causes a decrease in variable B; 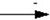 negative causal influence–other things being equal, an increase in variable A causes a decrease in variable B, or a decrease in variable A causes an increase in variable B. Meaning of variables in < >: the variable is copied to avoid decluttering the image with arrows and it is defined elsewhere in the model. balancing feedback loop, reinforcing feedback loop).

Fig 2 shows the stock and flow diagram of insect-based food adoption in the Netherlands. The structure of the model from “Potential adopters” to “Potential tasters” represents the process of deciding to taste insect-based food, and it is the first step towards adopting insect-based burgers. It starts with external influence on “Potential adopters”. Commonly reported *external influences* in insect-based food literature are promotion efforts in the form of direct education campaigns, such as bug banquets [45, 46], together with other mass media promotional activities [16, 33]. They “seed” the system with “potential tasters from promotional activities”, who will spread the word-of-mouth (WoM) about an innovation [16, 47], and so will initiate the *internal influence*. The more people have a chance to learn about insects through external influence, the more they spread WoM among people who had no experience with the new food [10], which increases the “average familiarity of the population”. For internal influence to have effect, potential adopters need to be able to find the product in shops to decide to taste it for the first time, which is a frequently mentioned obstacle towards acceptance of edible insects [10, 46, 48, 49]. Furthermore, not every contact with “Potential adopters” will be fruitful. Consequently, the amount of “potential tasters from word-of-mouth” depends on the “strength of the word-of-mouth” variable.

Nevertheless, merely exposing people to such food does not imply they will decide to taste it, due to negative feelings associated with insect-based food [50]. Negative feelings associated with tasting unappealing food, such as insects, can be divided in three dimensions: expectations of bad sensory properties (distaste), expectations of harmful consequences (danger or risk), and disgust [45, 50, 51]. However, disgust is one of the major predictors of willingness not to taste [50, 52], representing the main “barrier towards tasting” insect-based food for the first time in the model. In practice, disgust can be altered by the visibility of insects in the food, since decreased visibility had a positive influence on reducing the barrier to trying insect-based food [10, 53, 54].

#### From potential tasters to adopters or rejecters

Fig 2 physically separates people who are willing to taste insect-based food in general (“Potential tasters”) from the ones who are likely to adopt insect-based burgers once they appeared on the market in the Netherlands (“Adopters”). To adopt a certain food, people need to taste it first [55]. Trying the food facilitates learning to like the food and can have an influence on overall adoption [10, 56] by increasing familiarity with a certain food through reduction of uncertainty [5]. Increasing the overall familiarity with insect-based food can result in future increased adoption likelihood [45, 57, 58]. Familiarity can influence sensory affective motivation [5], or the distaste barrier [50], and ideational and safety motivations [5], or danger and disgust barriers [51], towards food adoption. Increasing familiarity of the whole population with insect-based food (“average familiarity of the population”) may partially lead to lowering the barrier towards adopting it [50].

Achieving familiarity with food through a tasting experience enables a more accurate prediction of perceived sensory properties of food [56], which could decrease the chances of coming to wrong inferences about the product’s sensory characteristics (“average taste expectations)” [5]. On the other hand, exposure to food can increasing the “average food appropriateness”, which facilitates the change of culturally embedded assumptions about inappropriate foods such as insects [5]. Moreover, for adoption of an insect-based food to occur, it is important that the specific insect-based food product tastes good [49, 56, 57, 59]. Therefore, the “average sensory quality of insect-based burger” directly influences the adoption rate and an extremely negative tasting experience may result in complete rejection of the food [5, 57]. Lastly, for adoption to be possible, it is necessary that the insect-based burger is available on the market. The variable “availability switch” allows the adoption of insect-based burgers only from the beginning of 2015, once they appeared on the market.

It is important to mention that the model is based on some assumptions that can notably affect the result of adoption. Firstly, it is assumed that people who have once tasted insect-based products will eventually decide towards adopting or rejecting such food. In reality, people might try edible insects multiple times before making the final decision, or even never make the decision. Furthermore, some people might never even taste insect-based food, for example, due to dietary restrictions (e.g., allergies). Secondly, the “average sensory quality of insect-based burger” is the average sensory liking estimated based on two studies. Schouteten et al. [59] performed research on sensory liking of insect-based burgers, and Tan et al. [5] studied ground beef and ground mealworm burgers. The shape and content of the products in these two studies was not the same, which could have made a difference in a respondent’s assessment, but the studies consistently reported low sensory liking. Lastly, some common marketing factors, such as influence of brand, competition, image or price are omitted from the model in Fig 2, since Sterman [16] recommends separating the flow of adoption from the flow of purchase.

Lastly, some variables have not been included in the model in Fig 2. Although advertising nutritional and environmental benefits of a product has been a frequent strategy to convince people into adopting them, it has been shown insufficient many times [5, 54, 60, 61]. Therefore, this strategy was not included in the model, which puts the focus on the taste of insect-based burgers [56, 59]. Moreover, food neophobia has been used in investigating acceptance of insect-based food. However, scientists have divided opinions regarding food neophobia and its direct influence on insect-based food acceptance. Tan et al. [5] claimed that food neophobia plays a minor role in the case of unusual foods such as insects. Hartmann et al. [57] stated that, regardless of food neophobia, the strongest predictor of willingness to eat insects was perceived taste. Nevertheless, food neophobia is indirectly present, since the model contains data on product appropriateness and taste expectations of consumers [5].

### Model formulation

After designing the stock and flow diagram, we developed equations based on the Bass diffusion SD model [16], literature on SD [16, 36, 43, 62], and data from reviewed literature on edible insects (see S1 Table). More specifically, existing empirical studies with Dutch consumers were employed, with a special focus on insect-based burgers studies, to confine the assumptions to only one social system. If no appropriate datasets were available for Dutch consumers, quantitative data were obtained from studies on Flemish consumers. Table 1 contains the equations of stocks, flows and auxiliary variables, together with their definitions, units and formulas, with the sources on which assumptions and adaptations were made. Table 2 lists the constant variables and their estimated values as used for the base run simulation (simulation with data extracted from and fitted to the literature sources).

**Table 1:** Stock, flow and auxiliary variables of the insect-based food adoption model–Vensim formulations and assumptions with sources*.

Variable	Definition	Unit	Formulation	Source	
**STOCKS**					
Potential adopters	People in the Netherlands who eat meat containing diets	people	= ∫-potential tasting rate dt + [Total population]	Formula adapted from [16]	
Potential tasters	People in the Netherlands who are likely to taste insect-based food	people	= ∫potential tasting rate—adoption rate—rejection rate *dt* + [0]	Formula adapted from [16]	
Adopters	People in the Netherlands who are likely to adopt an insect-based burger after tasting	people	= ∫adoption rate *dt* + [0]	Formula adapted from [16]	
Rejecters	People in the Netherlands who are likely to reject an insect-based burger after tasting	people	= ∫rejection rate *dt* + [0]	Formula adapted from [16]	
**FLOWS**					
potential tasting rate	Number of people tasting insect-based food for the first time per year	people/Year	= "potential tasters from word-of-mouth" + potential tasters from promotional activities	Formula adapted from [16]	
adoption rate	Number of people likely to adopt the product per year	people/Year	= Potential tasters*"likelihood to adopt insect-based food"*sensory quality adoption fraction*availability	Based on [51, 54, 57]	
rejection rate	Number of people likely to reject the product per year	people/Year	= (1-sensory quality adoption fraction)*Potential tasters*(1-"likelihood to adopt insect-based food")*availability	Based on [5, 50, 56]	
**AUXILIARY VARIABLES**					
average familiarity of the population	Average familiarity of the total population with insect-based food, based on the fraction of people who tasted it	Dmnl	= (Total population—Potential adopters)/Total population	Formula adapted from [16]	
barrier towards adopting	Average barrier towards adopting insect-based food, among the total population, as a sum of average taste expectation and average appropriateness, values from 0 to 1 (value 0 –full barrier; value 1 –no barrier)	Dmnl	= 1-(average taste expectation/2+average appropriateness/2)	Based on [5, 63]	
potential tasters from promotional activities	Number of potential adopters who are likely to taste insect-based food because of promotional activities each year	people/Year	= Potential adopters*barrier towards tasting*fraction of potential tasters from promotional activities	Based on [45, 46]	
potential tasters from word-of-mouth	Number of potential adopters who are likely to taste insect-based food as a result of word-of-mouth effect each year	people/Year	= (Potential adopters*"strength of the word-of-mouth"*average familiarity of the population)*barrier towards tasting	Formula adapted from [16]	
**AUXILIARY VARIABLES “WITH LOOKUP”**					
average appropriateness	Average appropriateness of insect-based food of the total population; based on average familiarity of the population with insect-based food	Dmnl	= WITH LOOKUP (average familiarity of the population, ([(0,0)-(1,1)], (0,0.01), (0.5,0.67), (1,0.72)))	Based on [5]
average taste expectation	Average taste expectation of insect-based food of the total population; based on average familiarity of the population with insect-based food	Dmnl	= WITH LOOKUP (average familiarity of the population, ([(0,0)—(1,1)], (0,0.28), (0.5,0.44), (1,0.56)))	Based on [5]
barrier towards tasting	Barrier towards tasting insect-based food as a result of average disgust levels of the population, from 0 to 1 (with assumptions: value 0 –full barrier; value 1 –no barrier)	Dmnl	= WITH LOOKUP (average disgust level, ([(0,0)—(1,1)], (0,1), (0.32,0.93), (1,0))	Based on [59]
likelihood to adopt insect-based food	Likelihood to adopt insect-based food, as a result of the barrier towards adopting, from 0 to 1 (with assumptions: value 0 –no barrier, full adoption; value 1 –full barrier, no adoption). Assumption is an averaged value of literature reported values.	Dmnl	= WITH LOOKUP (barrier towards adopting, ([(0,0)—(1,1)], (0,1), (0.55,0.12), (1,0)))	Based on [5, 54, 56]
sensory quality adoption fraction	Fraction of population adopting insect-based burger based on liking its sensory characteristics, from 0 to 1 (value 0 –no adoption; value 1 –full adoption)	1/Year	= WITH LOOKUP ("average sensory quality of insect-based burger", ([(0,0)—(1,1)], (0,0), (0.125,0), (0.6,0.5), (0.875,1), (1,1))	Based on [5, 49, 59]

*Model settings: time step: 0.125, integration type: RK4 Auto, units for time: Year.

**Table 2:** Constant variables of the insect-based food adoption model, with values used for the base run.

Variable	Definition	Unit	Formulation	Source
availability	Variable that represents availability of insect-based burgers in the Netherlands, with value 0 before year 2015 and value 1 from year 2015	Dmnl	= STEP (1, 2015)	Assumption based on [9]
average disgust level	The average level of disgust of the population when in the situation of tasting insect-based food, from 0 (no disgust, 100% chances of tasting) to 1 (100% disgust, 0% chances of trying)	Dmnl	0.32	Based on [57]
average sensory quality of insect-based burger	Average sensory liking of an insect-based burger and insect-based meatballs, normalized to 0–1 data range.	Dmnl	0.54	Based on [5, 59]
fraction of potential tasters from promotional activities	Fraction of potential adopters exposed to promotional activities of insect-based food	1/Year	0.0036	Assumption based on [15]
strength of the word-of-mouth	Probability that the contact with Potential adopters will result with fruitful word-of-mouth	Dmnl	= 0.151	Assumption based on [15]
Total population	Total model population representing people in the Netherlands expected to have meat eating diets. Based on the total population of the Netherlands in the year 2015 (16900720) minus 4% people with special eating habits (e.g. vegetarian, vegan, macrobiotic, anthroposophical)	people	16900720-(16900720*0.04)	Based on [64–66]

Most of the values of auxiliary and constant variables are based on theories and data grounded in the SD and edible insect literature. However, we made some assumptions, due to a lack of available data on insect-based food adoption. “Fraction of potential adopters from promotional activities” and “strength of the word-of-mouth” are variables with assumed values. These variables have an influence on the value of “average familiarity of the population”. They determine the number of people in the system who had the opportunity to taste insect-based food. The numerical values of these variables have been adjusted in such way that the system reaches a value of average familiarity of approximately 20% in the year 2015 [5]. Secondly, values of both variables were adjusted for the cumulative internal influence (number of “Potential tasters” in the end of the simulation as a result of internal influence) to be approximately ten times bigger than the cumulative external influence (number of “Potential tasters” in the end of the simulation as a result of external influence) [15].

## Model analysis

### Base run model simulation

**Fig 3:**
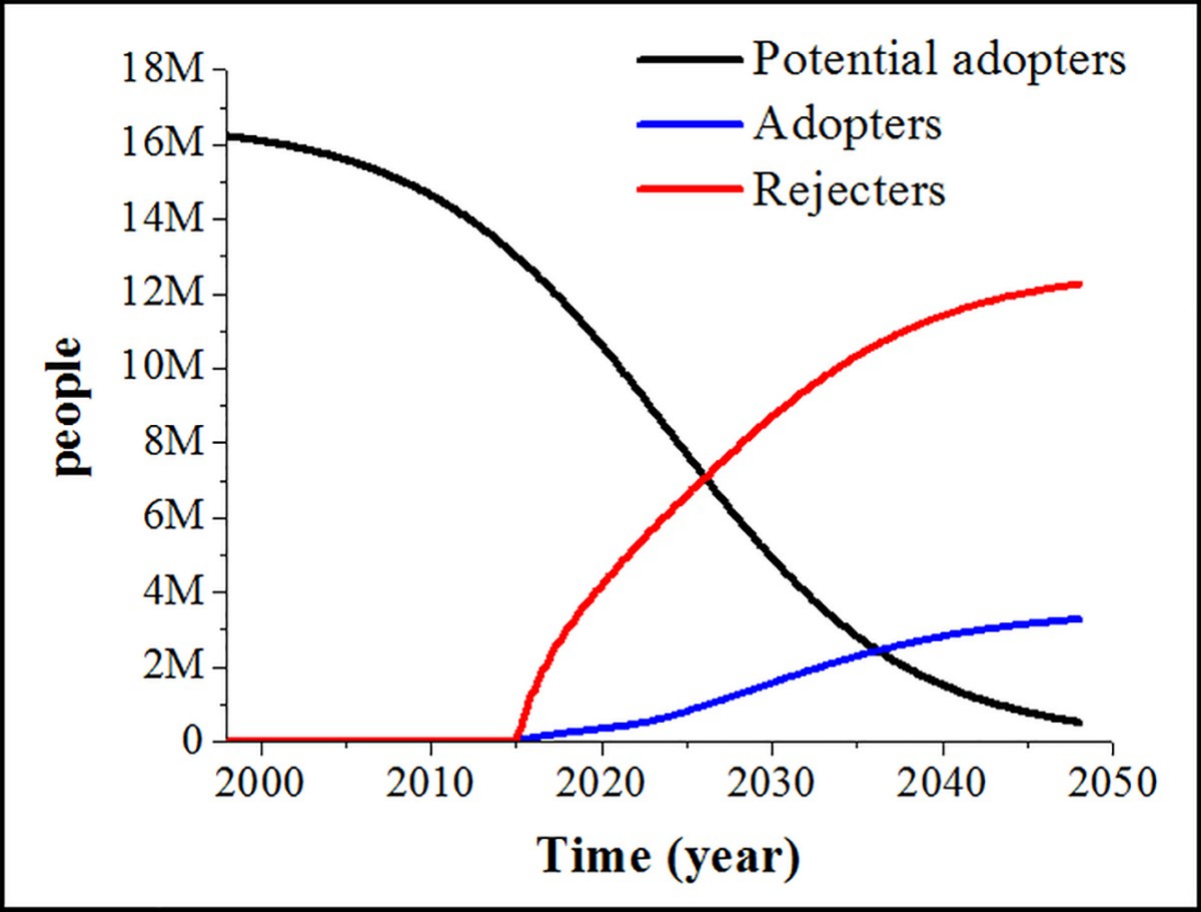
Base run model behaviour showing changes in the main stocks of the model on insect-based food adoption in the Netherlands.

Fig 3 represents the behaviour of the simulated model and it shows changes in the stock values from the year 1998 to 2048. Promotion of insect-based food in the Netherlands started in the late 1990s [67]. Since the exact year cannot be precisely determined, the year 1998 has been chosen to represent the start of the simulation. The model shows 50 years of insect-based food adoption, until the model base run approaches steady state. The same as the original Bass diffusion model [16], this model does not include population change in the Netherlands in the period from 1998 to 2048. This assumption was made for simplification reasons and is considered valid due to the qualitative interpretation of SD model results, where the exact values of stocks in the end of the model run are not as important as the shape of the curves [16].

Fig 3 shows that the diffusion process of insect-based food is slow in the beginning. The stock of “Potential adopters” depletes slowly. External influence, such as insect-based food promotional activities, is the main mechanism that drives depletion of the stock of “Potential adopters” in the beginning [16]. A fixed number of people could become familiar with insect-based food each year through these activities, which slowly increases the number of “Potential tasters”. “Potential tasters”, in turn, increase the chance of the word-of-mouth (WoM) adoption, by raising the variable “average familiarity of the population” and by increasing the probability of contact with “Potential adopters”. Internal influence, or the effect of WoM, gains strength over time and becomes the main adoption mechanism, contributing to the increased speed of depletion of the stock of “Potential adopters” from around year 2010 [16]. As the stock of “Potential adopters” depletes towards the year 2048, both mechanisms lose on their strength because of a lack of new “Potential adopters” in the system. Almost everybody is familiar with insect-based food by the year 2048.

Once insect-based burgers become available on the market, stocks of “Adopters” and “Rejecters” start filling with people who are familiar with insect-based food and who tasted insect-based burgers. A high number of people in the stock of “Rejecters”, and a low number of people in the stock of “Adopters” at the end of the simulation, show that the overall adoption is not very high. Adoption is partially influenced by “likelihood to adopt the product”, which is a result of “average taste expectation” and “average appropriateness” of insect-based food among the population, as experienced in previous tasting opportunities. The higher the average familiarity of the population is, the higher is the likelihood to adopt such food. Although an increase in average familiarity has a positive influence on the overall likelihood to adopt [5], its value is low throughout the whole simulation, which shows deeply embedded barriers towards insect-based food [63, 68].

Moreover, on top of the low likelihood to adopt, rejection based on “average sensory quality of insect-based burger” is a second challenge of the adoption process. At this point, sensory properties of insect-based burgers are still not competitive to the meat-containing burgers, which are usually the reference point when deciding upon such a product’s taste [59]. Positioning insect-based food as meat substitutes, such as an insect-based burger, reinforces consumers to compare them with meat products, expecting them to taste similar [60, 69]. Tasting an insect-based product that is supposed to correspond to a meat alternative can result in an unsatisfactory sensory experience. Consequently, after the initial negative tasting experience, most of the “Potential adopters” reject the innovation.

The model base run demonstrated that diffusion of radical innovations, such as insect-based burgers, takes a long time, as many years must pass before there is a high rise in the number of “Adopters”. The diffusion process of insect-based burgers among the Dutch population started only recently and many years may pass before all Dutch consumers become aware of such foods, under the assumption that insect-based food will continue being available and promoted.

### Analysis of scenarios

We proposed several simple scenarios (Table 3) to study the influence of change in various model variables on model behaviour, bearing in mind that the variables we changed can be influenced in real life, for example when developing launch strategies. Each of the scenarios targets only one or two model variables, to establish a clear connection between the parameter change and the emergent model behaviour.

**Table 3:** Description of the three different scenarios as simulation experiments.

Scenario	Description	Value or formula (unit) of the changed variable
**Scenario 1 (s1) Internal and external influence**	Scenario 1 tests the effect of increase in internal and external influence, compared to the base run. In the first case (s1.1), only the value of the variable “strength of the word-of-mouth” increases for 10% from the year 2015. In the second case (s1.2) both “strength of the word-of-mouth” and “fraction of potential tasters from promotional activities” are increased (10% and 100% respectively) from the year 2015 (when insect-based burgers became available on the Dutch market).	**(s1.1)** “strength of the word-of-mouth” = 0.151 + STEP (0.151*1.1, 2015) (1/Year)
		**(s1.2)** “fraction of potential tasters from promotional activities” = 0.0036 + STEP (0.0036*2, 2015) (1/Year); “strength of the word-of-mouth” = 0.151 + STEP (0.151*1.1, 2015) (1/Year)
**Scenario 2 (s2) Sensory quality improved**	Scenario 2 tests the effect of gradual (s2.1) and immediate (s2.2) increase in sensory quality of insect-based burgers, compared to the base run. In the first case (s2.1), the value of the “average sensory quality of insect-based burger” variable increases linearly from the year 2017 until 2048. In the second case (s2.2), it increases immediately.	“average sensory quality of insect-based burger” = **(s2.1)** linear growth from 0.54 (Dmnl) in year 2017 to 0.8 (Dmnl) in year 2048
		**(s2.2)** 0.8 (Dmnl) from the year 2017
**Scenario 3 (s3) Likelihood to adopt increased**	In scenario 3, the effect of changed “likelihood to adopt insect-based food” variable on adoption rate is tested. Instead of only comparing it to the base run, we employed what has been learned in previous scenario. We increased the variable “likelihood to adopt insect-based food” (s3.1) and compared it to the base run, and to the model run when both likelihood to adopt and sensory quality are increased (s3.2)	**(s3.1)** “likelihood to adopt” = WITH LOOKUP (barrier towards adopting, ([(0,0)—(1,1)], (0,1), (0.55,0.19), (1,0)) (Dmnl)
		**(s3.2)** variables changed according to S3.1 and S2.2

**Fig 4:**
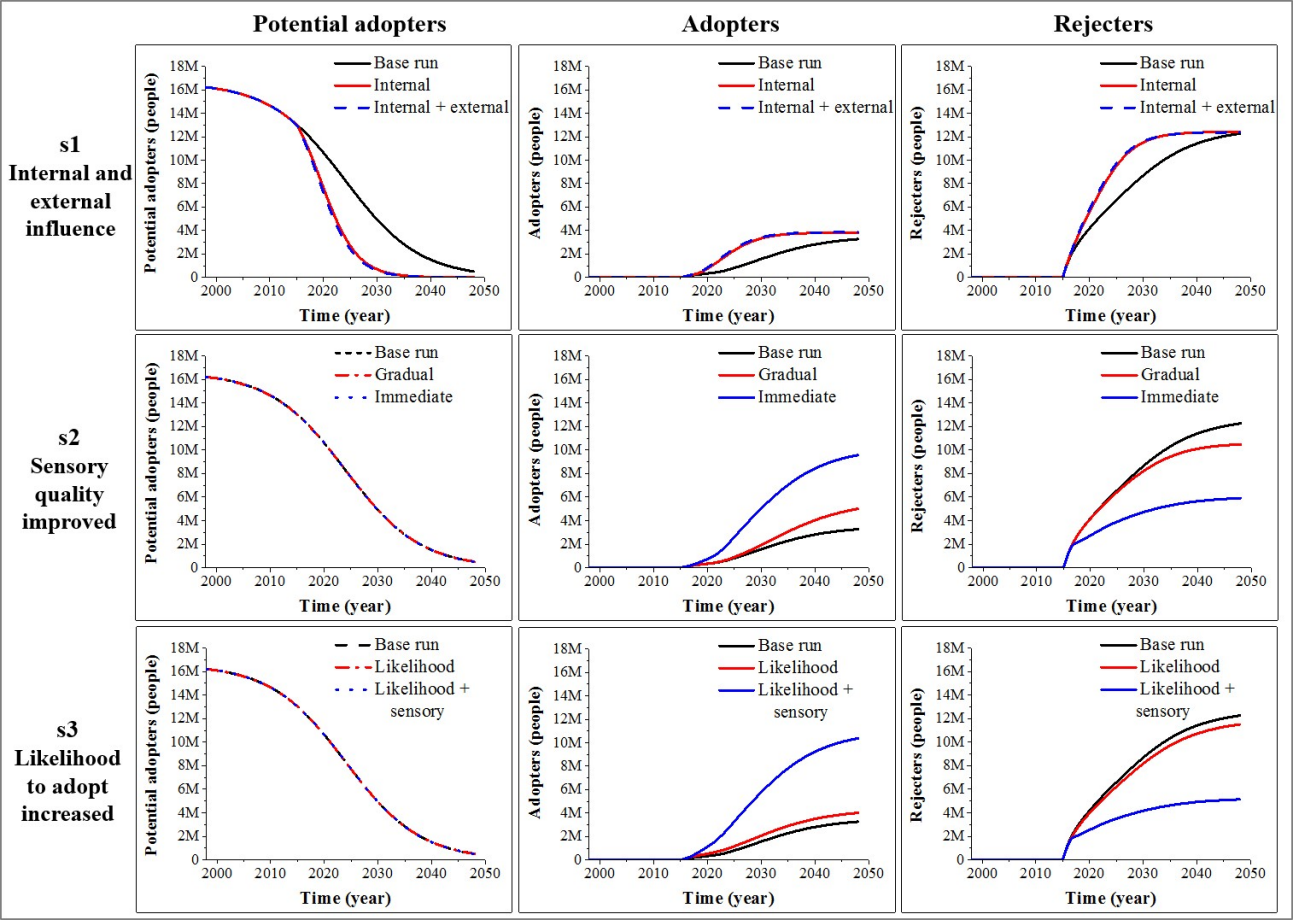
Behaviour of “Potential adopters”, “Adopters” and “Rejecters” stocks for 3 different scenarios (scenario 1 (s1): Increase in internal and external influence; scenario 2 (s2): Increase in sensory quality; scenario 3 (s3): Increase in likelihood to adopt).

In scenario 1, we analysed the effect of an increase of internal (WoM) and external influence (promotion) on adoption rate and on the overall adoption outcome, compared to the model base run. Fig 4 contains the results of the analysis. Due to the low sensitivity of the variable “fraction of potential tasters from promotional activities” (see S1 Fig in S1 File), we only displayed the effect of doubling the variable “strength of the word-of-mouth” (internal influence, s1.1), and of both increased promotional activities and WoM (s1.2). The increased WoM (s1.1) raises the rate with which the stock of “Potential adopters” depletes, compared to the base run, which leads to a faster tasting rate, and to a faster adoption rate (see the increase in the stock of “Adopters”). However, both adoption outcomes are similarly low in both s1.1 and s1.2. The problem lies partially in the low sensory quality of insect-based burgers and in the high barriers towards adopting insect-based food (“average taste expectation” and “average appropriateness”), which are the direct cause of low “likelihood to adopt”. Van Huis et al. [48] stated that such barriers are deeply embedded and are not easy to change, especially with people highly sensitive to animal reminder disgust [45]. “Animal-reminder disgust is based on reminding people of their own animal nature” and mortality, e.g., injuries to the body or death [45, pg. 320; 70]. Furthermore, when both the internal and external influence are increased (s1.2), there is almost no change in adoption rate, compared to scenario s1.1. Promotional activities may have a somewhat positive impact on willingness to try unusual foods in future situations [46, 48]. However, their long-term influence is weak, as such promotion cannot reach many people [45]. Nevertheless, promotion is a relevant ingredient towards wider familiarity in the beginning of the adoption process, and consequently, towards consumption of insects [10]. It provides people who “seed” the population and initiate WoM [16], but it should not be the only strategy when the main goal is adoption of insect-based food.

In scenario 2, we studied the effect of increased sensory quality on model behaviour (s2.1 and s2.2), compared to the base run behaviour. Firstly (s2.1), we assumed that the improvement in sensory quality of burgers will be gradual, and the value of the variable “average sensory quality of insect-based burger” will increase linearly from the year 2017 until 2048. In the second case (s2.2), we wanted to explore adoption of a very tasty insect-based burger already from the year 2017, which is a product that has not yet been reported in scientific literature (*e*.*g*., [59, 71, 72]). Contrary to the first scenario, there is an increase in the overall adoption in both cases. Gradual improvement in the sensory quality of insect-based burgers could lead to a faster adoption rate and increased overall adoption. This effect is even more emphasized in the case of immediate improvement in sensory quality, which could lead to even greater overall adoption among Dutch population after 50 years of diffusion. However, due to, once again, barriers towards adopting, there could still be a high number of rejecters. High sensory liking is not sufficient for incorporating an insect-based product into regular consumption [71]. Scenario 2 indicates that improvement in sensory quality of insect-based products is an urgent need. The longer people are exposed to products that are not tasty, the more cumulative rejecters there are. Assumingly, those people might decide never to taste insect-based products again, even if the products become tasty in the future. Consequently, making available insect-based food that is not yet of high sensory quality is a strategic decision that can hinder broader consumption of such food in the long run.

Finally, in scenario 3, we analysed how a change of the variable “likelihood to adopt insect-based food” affected model behaviour. In edible insects literature, the likelihood to adopt has been assessed through willingness to eat (*e*.*g*., [73]) or readiness to adopt (*e*.*g*., [54]), with substantial differences in reported quantitative values. Here (s3.1), we chose one of the higher reported values (*e*.*g*., [54]) to compare simulation results to the base run, but also to the situation of an immediate increase in sensory quality of insect-based burgers in s3.2. The value of the “likelihood to adopt insect-based food” variable in s3.1 and s.3.2 is 1.6 times bigger than in the base run. Fig 4 shows that changing the value of “likelihood to adopt” in s3.1 can have an effect on increasing the adoption outcome, however, much less than 1.6 times. The “barrier towards adopting”, resulting from unfamiliarity and expectations of negative taste, and negative appropriateness, directly affects the likelihood to adopt [5, 63]. To observe a substantial change in adoption, there is a need for combining an increase in adoption likelihood with an immediate increase in sensory quality of the product, as in s3.2. Scenario 3 shows the importance that past experiences with insect-based food have on adoption of such food [56, 61]. Beliefs acquired from past experiences do not change fast. However, introducing immediate positive incentives, such as high sensory quality, can move adoption in a beneficial direction.

Scenarios 2 and 3 did not influence the “potential tasting rate”, which is visible from the unchanged behaviour of the stock of “Potential adopters”. One of the limitations of the Bass diffusion model is that it does not assume the influence of negative WoM. Nevertheless, even with this limitation, it has been recognized that such models capture historical behaviour very well [16]. In the future, it would be necessary to explore mechanisms and obtain dynamic datasets to further study the influence of this limitation on model behaviour. For example, the “strength of the word-of-mouth” could be affected by positive and negative WoM, according to changes in the amount of “Adopters” and “Rejecters”. Moreover, due to the narrow boundary, the model does not capture the real situation in the marketplace. It shows only the number of people who would be willing to adopt insect-based burgers, while it does not indicate the sales of such products.

## Discussion

**Fig 5:**
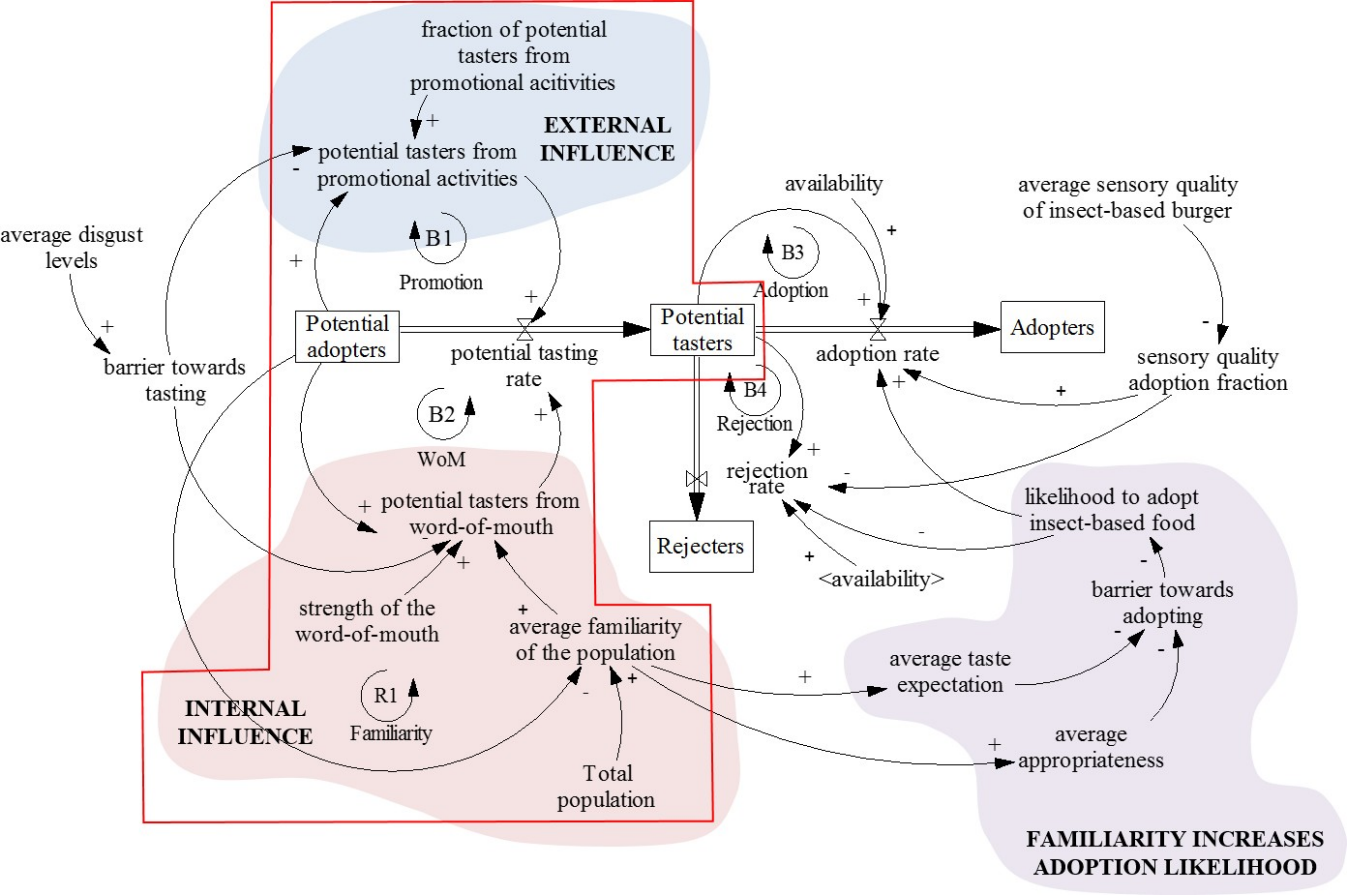
Boundary of the adapted SD Bass diffusion model and the original SD Bass diffusion model (the part of the stock and flow diagram inside the red lines).

In this paper, we aimed at extending an existing SD Bass diffusion model to develop and simulate adoption of a radical new food product, i.e. an insect-based burger. Fig 5 graphically demonstrates the extended boundary of our model, compared to the original SD Bass diffusion model (e.g., [16]). The extended SD Bass diffusion model offered a basis to explore insect-based food diffusion as a complex dynamic problem, by identifying cause-effect relationships among major variables, and by examining the outcome of diffusion over a longer time horizon [5, 16, 24, 25, 36]. Instead of focusing on ‘detail complexity’ of food choice, i.e. the quantity of model components, we aimed at studying ‘dynamic complexity’, i.e. the behaviour over time that the model produces [16]. By grounding the literature review in DoI and the Bass diffusion SD model, we were able to only select variables from existing literature that are relevant to represent consumer adoption over time as a dynamic complex problem, and to present them in the form of a stock-and-flow diagram.

The model facilitated exploring the extent to which current literature on edible insects supports studying consumer adoption over time. We were able to identify mechanisms that require further investigation, and the data that is needed to increase the understanding of insect-based food adoption. For example, effects such as negative word-of-mouth, and adoption of insect-based burgers by people who initially reject them were not included in the model, while they were also omitted in the original Bass diffusion model [16]. Moreover, they have not been studied in the literature on edible insects. Furthermore, the effect of price has rarely been researched in insect-based food literature [20], and we could not fully include it in the model in Fig 2 due to a lack of appropriate data and clarity of mechanisms. These identified knowledge gaps are potential future consumer research opportunities.

Confining the model boundary to DoI and the Bass diffusion SD model imposed certain trade-offs that affected the level of detail with which consumers are presented in the model. Consumers’ decisions are a result of complex individual psychological processes, and SD does not fully allow inclusion of variations among individual consumers in a social system. SD models belong to a group of aggregate models where consumers are studied as a perfectly mixed collection of individuals with an aggregated behaviour, instead of representing individual behavioural characteristics of consumers [15, 16, 39]. Separating consumers into different groups based on socio-demographic characteristics could be one of the future model improvements, after appropriate data collection. If one aims at modeling the micro-level of consumer behaviour and at capturing individual psychological consumer characteristics, another computer modelling method would be more appropriate, for example, agent-based modelling [16, 74].

The system dynamics approach has rarely been used to study topics related to processed food products. One example is the model developed by Zhao and Zhong [75], who explored the effect of carbon labelling of milk on the occurrence of ordinary and loyal customers of such products. Their model focused on extrinsic product characteristics (i.e., the characteristics communicated on the packaging) affecting food choice and it involved variables related to environmental aspects of food product attributes. The SD model presented in this paper is the first example of employing the SD Bass diffusion model to study adoption of radical foods. In our model, based on existing literature on insect-based foods, the focus is on aspects of consumer food choice such as sensory quality, disgust and food appropriateness, which can be altered by adjusting intrinsic product characteristics (e.g., taste, texture). For example, by using grinded instead of whole insects, consumers experience less disgust, which can have a positive impact on sensory quality and food appropriateness perception [56]. To estimate future trends in adoption of conventional and radical foods, both extrinsic and intrinsic product characteristics, together with other marketing mix aspects, should be represented by model variables. However, this should also be supported by an appropriate data collection, which is currently a limiting factor in the case of insect-based burgers. Data scarcity in the edible insects field has been recognized earlier by a few authors [e.g., 20, 73] and it could be a sign of a limited view to solving the insect-based food adoption problem [16].

The current model was developed to showcase the SD modeling and simulation approach as an opportunity towards broader utilization of modelling in studying complex dynamic problems in food science and consumer research. The model is suitable for understanding the extent to which current practices, under the proposed framework, affect the rate of insect-based food adoption. However, it is not suitable for understanding if people will buy a product in a market environment. Instead of predicting the exact number of adopters of edible insects, it serves as an example of a method to develop “white box” models in consumer research. Commonly used empirical modeling approaches do not consider underlying mechanisms, and seek simplified relationships to correlate variables, while “white box” models aim at representing those mechanisms [76]. Similar SD consumer models could have a standard use in new food product development, once enriched with data that would more closely mimic the marketplace situation. Consumers are changing [77], and food companies need to be able to develop optimal product strategies fast. Using SD models prior to launching new products could help product managers in their decision-making, by testing the consequences of their actions on consumer product adoption over time. SD models could also serve the purpose of steering future marketing and consumer research, after discovering model variables that substantially affect model behaviour. Moreover, similar SD models could be used as learning tools, by giving managers a chance to learn from experimenting with the model.

The initiative for consumption of edible insects in Western countries comes from a need to reduce livestock protein consumption to decrease negative environmental impact of the livestock sector [78]. According to Kim et al. [79], foods of animal origin contribute to 18% of calorie and 25% of protein intake worldwide. Moreover, due to the growth of the population, an increase of 57% in global demand for meat between 2005 and 2050 was projected [79]. Insects are a promising source of nutrients for humans and their production results in lower greenhouse gas emissions [80, 81]. Therefore, the topic of insect-based food consumption is not only relevant for companies that develop such products, but also for governments. The SD model in this study suggested a low adoption of an insect-based burger in the Netherlands under currently reported practices, which can imply that similar products might also have a low adoption. There is a need to evaluate which government policies might positively impact future consumption of edible insects and how. The SD approach can be beneficial in that respect and similar models could facilitate uncovering potentially successful governmental policies, which would reinforce a widespread consumption of more sustainable sources of protein such as insects. However, existing literature on edible insects does not address the role of governments in supporting adoption of edible insects, and more research is needed to uncover which policies could be implemented in the future. In the past, the SD approach has been employed to study topics of public relevance related to food consumption, such as obesity (e.g., [82]). In the future, SD models could also be used to examine which public initiatives could potentially lead to more sustainable food consumption.

### Main contributions of this study

#### Extension of the original system dynamics Bass diffusion model to study adoption of food products

**Fig 6:**
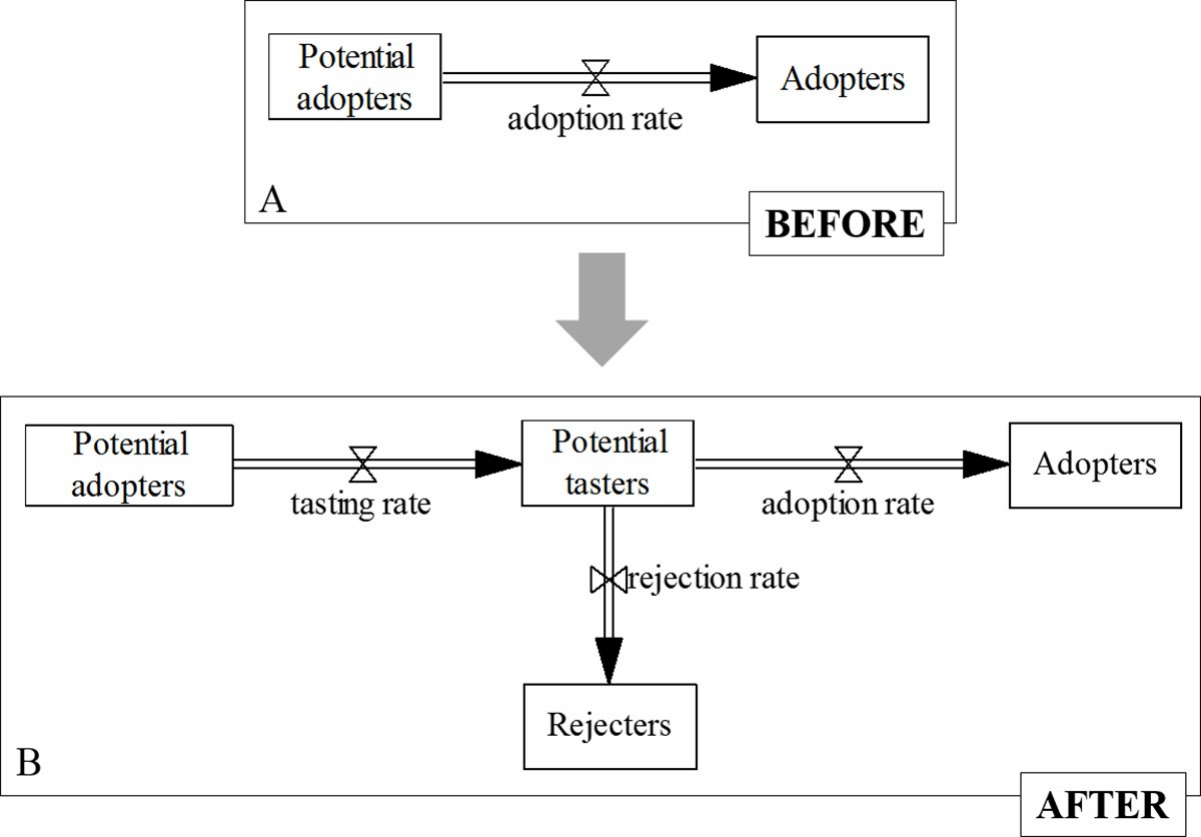
Contribution of this study–an extension of the original SD Bass diffusion model. (6A) Original SD Bass diffusion model; (6B) an extended SD Bass diffusion model to study adoption of food products.

This study represents the first example of an SD Bass diffusion model to study adoption of radical food products. Although a wide range of adoption studies based on the original SD Bass diffusion model exist (*e*.*g*., [26–30]), these models were not appropriate to represent adoption of a food product. The original SD Bass diffusion model was extended by adding two additional stocks, “potential tasters” and “rejecters”, to capture specific elements inherent for adoption of new food (see Fig 6). Therefore, in the case of adoption of food products, “potential adopters”, if they are influenced by advertising or WoM, will first become “potential tasters” (Fig 6B). Furthermore, our SD model has a stock of “rejecters”, who do not adopt the product due to reasons such as low sensory quality, likelihood to adopt or availability.

#### Extension of the analytical Bass diffusion model to study adoption of food products

The adapted SD Bass diffusion model can be express in a mathematical form, which is a contribution to analytical modelling of food product adoption:

$$\frac{dN(t)}{dt}=c(t)T(t),$$ (2)

$$\frac{dT(t)}{dt}=(p_{2}+\frac{q_{2}}{m}(T(t)+R(t)+N(t))(m‐T(t)‐R(t)‐N(t))‐c(t)T(t)‐d(t)R(t),$$ (3)

where p_2_ is the coefficient of innovation, which was in this case fraction of tasters from promotional activities multiplied by barrier towards tasting, q_2_ is the coefficient of imitation, which was in this case strength of the word-of-mouth multiplied by barrier towards tasting, m is the total population or potential adopters, T(t) is the number of potential tasters in time t, R(t) is the number of rejecters in time t, N(t) is the number of adopters at time t, m is the total population or potential adopters, c is the coefficient of adoption, which was in this case the multiplication of availability, sensory quality adoption fraction and likelihood to adopt insect-based food, and d is the coefficient of rejection, which was in this case multiplication of availability, (1-sensory quality adoption fraction) and (1-likelihood to adopt insect-based food).

#### Model simulation results as tools to develop strategies for improving insect-based food adoption

Simulations revealed that the diffusion of insect-based food, such as an insect-based burger, will be a long process under the currently reported practices in the Netherlands. In scenario 1, we analysed the effect of increase of word-of-mouth and promotion on the adoption rate. While promotion is important to increase the initial familiarity of the population with such products, the main mechanism that can strongly affect diffusion of such food is word-of-mouth, occurring after a tasting experience. In scenario 2, we studied the effect of increased sensory quality on the adoption rate, and in scenario 3, we studied the effect of increased likelihood to adopt. The simulations revealed that to observe a substantial change in adoption under increased adoption likelihood, there is a need of combining it with an increase in sensory quality of the product. To increase likelihood to adopt, there is a need to increase average familiarity of the population with insect-based food, to improve consumers’ taste expectations and perception of appropriateness of such food.

## Conclusions

In this study, we developed an extended SD model of insect-based food adoption in the Netherlands, based on current literature on edible insects. This is the first application of the SD approach to study the complex problem of adoption of radical new foods from a dynamic perspective, and it aimed at showcasing an example of developing a “white box” model for use in food science and consumer research.

The extended SD Bass diffusion model allowed representation of current findings in literature on edible insects in the form of an aggregate stock-and-flow model, which offered a means of experimenting with various adoption strategy scenarios. The base run demonstrated that the diffusion process in this case has been slow. The main learning outcome was that the internal model structure we developed can explain the adoption process of insect-based food. The main mechanism that can strongly affect diffusion of such food is the word-of-mouth. Therefore, the advice for the future would be that attention needs to be given to triggering consumers to communicate their positive experiences with tasting insect-based food to people who have not tasted such food.

Moreover, based on current literature on edible insects, we could build an SD of a narrow boundary. More concretely, past studies on insect-based food mainly explored factors related to consumer psychology of choosing insect-based food, which were the basis for our model. However, multiple other stakeholders influence the process of food adoption, such as food companies competing on the market and governments setting regulations. This could indicate that, currently, the problem of insect-based food adoption is tackled from a narrow perspective, which could be an obstacle towards developing successful strategies for adoption of insect-based food.

Furthermore, SD was beneficial in uncovering knowledge gaps that can guide future research. Based on our findings, we suggest that future consumer research on insect-based food should be performed by separating research on willingness to taste food for the first time from research on willingness to adopt certain food, since they are guided by different mechanisms. Furthermore, mechanisms related to positive and negative word-of-mouth, and a possibility of adoption of insect-based burgers by people who initially reject them should be addressed. Also, the influence of factors like availability, strength of the word-of-mouth, and promotional activities on adoption should be addressed by collecting more specific data. Furthermore, longitudinal research, in which consumers would be observed throughout longer time, should be established, to provide data for the future development of similar models. Further improvement of this SD insect-based food adoption model could be made by collecting data from other sources (*e*.*g*., sales data from retailers or food companies) and by expanding the scope of the model *(e*.*g*., to include marketing mix elements, influence of government incentives, and technological development), and by collecting sales data of existing insect-based food to assess model’s forecasting power. Lastly, future research could move towards employing other simulation modelling methods that can be used for dynamic complex problems, or by combining them with SD. For example, in agent-based models, consumers are represented as individual agents, instead of in an aggregated way. Such a model can stand alone or be an addition to an SD model.

## Supporting information

S1 File (DOCX)

S1 Dataset (ZIP)

S1 Table Data used to formulate variables of the insect-based food adoption model. (DOCX)
