# Supplementary material for: Modifying the Bass diffusion model to study adoption of radical new foods–The case of edible insects in the Netherlands
Source: PLoS One. 2020 Jun 11;15(6):e0234538. doi: 10.1371/journal.pone.0234538 (PMC7289433; doi:10.1371/journal.pone.0234538)
Supplement: S1 File — (DOCX) [file pone.0234538.s001.docx]

Supplementary material 1:

Structure oriented behaviour tests of model validation

Structure oriented behaviour tests, with the purpose of model validation, included sensitivity analysis and extreme conditions tests [1-3]. This supplementary material contains examples of the two performed tests.

Sensitivity analysis

Sensitivity analysis includes studying the effect of small changes in parameters on model behaviour in order to look for model errors, to understand the relationships between inputs and emergent behaviour, and to identify highly sensitive inputs [3, 4]. The aim is to understand if performed changes lead to, for example, behaviour sensitivity (changes in modes of behaviour), or to mere numerical sensitivity, and to what extent [3]. This helps with establishing an order of preference in policies, or pinpoints to sensitive variables that need to receive greater attention when estimating the values and formulas.

We studied the effect of 10% change in constant variables. All simulations were performed with Sensitivity Control feature of Vensim DSS, Version 6.4b [5]. The following settings were employed: multivariate sensitivity simulations (allows for Monte Carlo simulations); number of simulations: 1000; noise seed: 1234; distribution: random uniform; minimum value: base run parameter value-10%*base run parameter value; maximum value: base run parameter value+10%*base run parameter value.

We will demonstrate the results of sensitivity analysis for two variables, to display different sensitivity levels, i.e. “fraction of potential tasters from promotional activities” and “average sensory quality of insect-based burger”. Table 1 shows the variable values used for the sensitivity analysis. Sensitivity analysis was performed separately for each variable. The same approach has been used for all the other constant variables in the model.

Table 1: minimum and maximum values of variables used for sensitivity analysis

| Variable | Base run value (Dmnl) | Minimum value (Dmnl) | Maximum value (Dmnl) |
| --- | --- | --- | --- |
| fraction of potential tasters from promotional activities | 0.0036 | 0.00324 | 0.00396 |
| average sensory quality of insect-based burger | 0.54 | 0.486 | 0.594 |


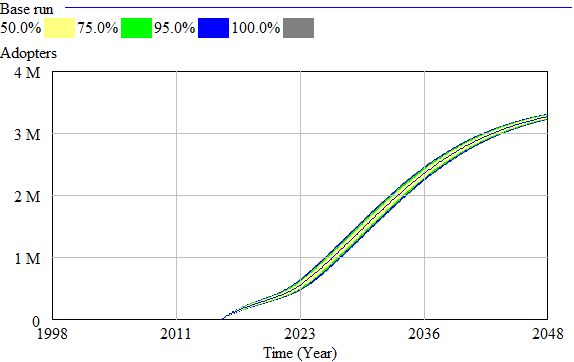


**Fig 1**: results of the sensitivity analysis of the variable “fraction of potential tasters from promotional activities”


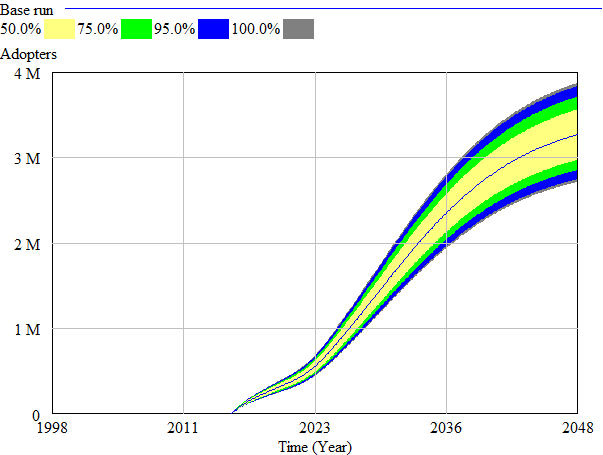


**Fig 2**: results of the sensitivity analysis of the variable “average sensory quality of insect-based burger”

Results of the sensitivity analysis in Fig 1 and Fig 2 are shown as graphs with confidence bounds. Graphs are assessed qualitatively, by asking if the real system would be similarly sensitive to the tested parameters. One aims at determining the parameters to which the model is highly sensitive [4]. Figs 1 and 2 show that the behaviour of the stock “Adopters” is not strongly sensitive to the variable “fraction of potential adopters from promotional activities”, while the variable “average sensory quality of insect-based burger” is numerically sensitive. Although the aim of the modelling process is not to predict the outcome of the diffusion process, but to study the effect of changes on model behaviour, the outcome of sensitivity analysis may have implications for future data collection. To have more confidence in the results, one would need to assess sensory quality variable with more precision than the other tested variable. Furthermore, Fig 2 suggests that both variables do not express behaviour sensitivity, as they both show S-shaped growth. This implies that the model behaviour results from the model structure, and not from the uncertainty of the variables.

Extreme conditions test

Extreme conditions test includes evaluation of model equations under extreme conditions [1]. It is performed by assigning extreme values to input variables to assess if the model responds plausibly. The aim is to test if the model conforms to the basic physical laws (*e.g.* if there is no product available, there will be no adopters) [2]. Although we here give only one example, this test has been performed on other input variables in the same manner.

We will demonstrate an example of extreme conditions test on the variable “fraction of potential tasters from promotional activities”. To assess if the model responds appropriately to extreme values, we assigned value zero to the variable “fraction of potential tasters from promotional activities”, assuming a situation where there is no external influence to seed the system with tasters who can start spreading word-of-mouth. Fig 3 shows that the model passed the extreme conditions test since there are no adopters.


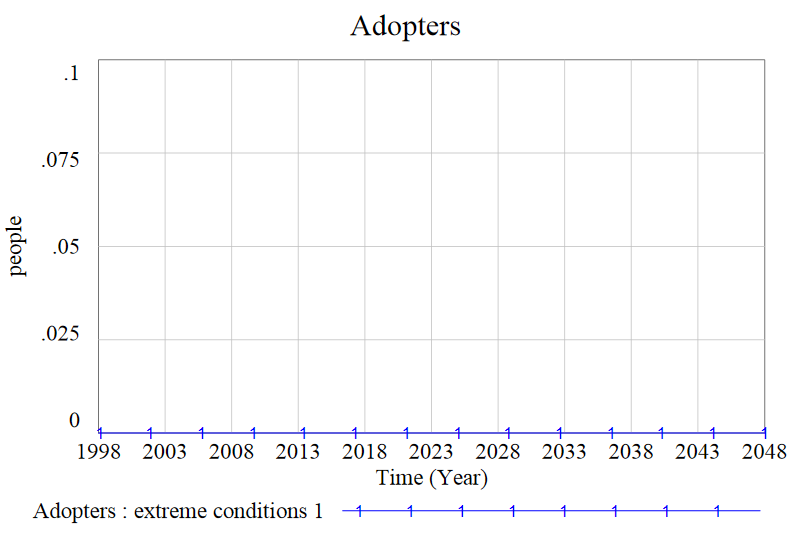


**Fig 3**: result of extreme conditions test for variable “fraction of potential tasters from promotional activities”

References:

1. Barlas Y. Model validation in system dynamics. In Monaghan C, Wolstenholme E, editors. Proceedings of the 12th International Conference of the System Dynamics Society; 1994; Stirling. p. 1-10.
2. Sterman JD. Business Dynamics: Systems Thinking and Modelling for a Complex World (International Edition). Singapore: McGraw-Hill, 2004. 982 p.
3. Pruyt E. Small System Dynamics Models for Big Issues: Triple Jump Towards Real-World Dynamic Complexity. Delft: TU Delft Library; 2013. 317 p.
4. Barlas Y. Formal aspects of model validity and validation in system dynamics. Syst Dyn Rev. 1996;12(3): 183-210.
5. Vensim DSS. Harvard: Ventana Systems, Inc.; 2018.
