# Supplementary material for: Modifying the Bass diffusion model to study adoption of radical new foods–The case of edible insects in the Netherlands
Source: PLoS One. 2020 Jun 11;15(6):e0234538. doi: 10.1371/journal.pone.0234538 (PMC7289433; doi:10.1371/journal.pone.0234538)
Supplement: S1 Dataset — (ZIP) [file pone.0234538.s002.zip › Data submission/How to open the files.docx]

**Installing Vensim software**

Vensim is a software for using and developing system dynamics models, and it also has a free version.

To install the free Vensim software, visit the following webpage:

<http://vensim.com/free-download/>
