# Supplementary material for: Modifying the Bass diffusion model to study adoption of radical new foods–The case of edible insects in the Netherlands
Source: PLoS One. 2020 Jun 11;15(6):e0234538. doi: 10.1371/journal.pone.0234538 (PMC7289433; doi:10.1371/journal.pone.0234538)
Supplement: S1 Table — (DOCX) [file pone.0234538.s003.docx]

S1 Table

Table 1: Data used to formulate variables of the insect-based food adoption model

| **Variable** | **Definition** | **Data used from the source(s)** | **Source*** | |
| --- | --- | --- | --- | --- |
| average appropriateness | Average appropriateness of insect-based food of the total population; based on average familiarity of the population with insect-based food | Evaluation of expected appropriateness within three familiarity levels:  Tasted before: 6.5/9  Known as food, never tasted:6/9  Not known as food:3.2/9 | Based on figure 2 from [5] |  |
| average taste expectation | Average taste expectation of insect-based food of the total population; based on average familiarity of the population with insect-based food | Evaluation of taste expectation within three familiarity levels:  Tasted before: 5.2/9  Known as food, never tasted:4/9  Not known as food:2.5/9 | Based on figure 2 from [5] |  |
| barrier towards tasting | Barrier towards tasting insect-based food as a result of average disgust levels of the population | [5] 98.97% participants tasted a mealworm burger  [59] 88.68% of the study participants did not want to try an insect-based burger | Based on [5, 59] |  |
| likelihood to adopt insect-based food | Likelihood to adopt insect-based food, as a result of the barrier towards adopting | [54]: 19.3% ready to adopt insects as a meat substitute  [73]: 5% consumers ready to adopt | Based on [54, 73] |  |

| availability | Variable that represents availability of insect-based burgers in the Netherlands, with value 0 before year 2015 and value 1 from year 2015 | evidence of insect-based burgers being present on the Dutch market in 2015 | Based on [9] |
| --- | --- | --- | --- |
| average disgust level | The average level of disgust of the population when in the situation of tasting insect-based food, from 0 (no disgust, 100% chances of tasting) to 1 (100% disgust, 0% chances of trying) | 32% of participants mentioned disgust as an emotion related to insect-based burgers | Based on table 3 from [59] |
| average sensory quality of insect-based burger | Average sensory liking of an insect-based burger and insect-based meatballs | [5] Average sensory liking: 6/9  [59] Average sensory liking: 4.2/9 | Based on [5, 59] |
| fraction of potential tasters from promotional activities | Fraction of potential adopters exposed to promotional activities of insect-based food | Calibrated for the model to reach average familiarity of approximately 22% by 2015 [5], and for cumulative internal influence (number of “Potential tasters” in the end of the simulation as a result of word-of-mouth) to be approximately ten times bigger than cumulative external influence (number of “Potential tasters” in the end of the simulation from promotional activities)[15]. | Based on [5, 15] |
| strength of the word-of-mouth | Probability that the contact with Potential adopters will result with fruitful word-of-mouth | Calibrated for the model to reach average familiarity of approximately 22% by 2015 [5], and for cumulative internal influence (number of “Potential tasters” in the end of the simulation as a result of word-of-mouth) to be approximately ten times bigger than cumulative external influence (number of “Potential tasters” in the end of the simulation from promotional activities)[15]. | Based on [5, 15] |
| Total population | Total model population representing people in the Netherlands expected to have meat eating diets | [64] Total population of the Netherlands in the year 2015 was 16900720  [65, 66] 4% of the population with special eating habits (e.g. vegetarian, vegan, macrobiotic, anthroposophical) | Based on [64-66] |

*For references see the main text of the manuscript
